# 1054. Comparison of Post-Acute COVID-19 Symptoms in Infected Individuals Pre- and Post-Vaccination

**DOI:** 10.1093/ofid/ofac492.895

**Published:** 2022-12-15

**Authors:** Dylan McDonald, Jennifer Logue, Nicholas M Franko, Megan M Kemp, Denise J McCulloch, Eric J Chow, Helen Y Chu

**Affiliations:** University of Washington, Seattle, Washington; University of Washington, Seattle, Washington; University of Washington, Seattle, Washington; University of Washington, Seattle, Washington; University of Washington, Seattle, Washington; Public Health - Seattle & King County, Seattle, Washington; University of Washington, Seattle, Washington

## Abstract

**Background:**

A constellation of debilitating symptoms, known as post-acute sequelae of COVID-19 (PASC), has been described in those in those with prior SARS-CoV-2 infection. While SARS-CoV-2 vaccination remains an effective way to prevent severe illness, PASC in individuals infected after vaccination is not well characterized.

**Methods:**

A cohort of adults with laboratory confirmed SARS-CoV-2 infection were enrolled as cases and longitudinally followed between March 2020-March 2022 in the greater Seattle region. Demographic and acute illness surveys capturing baseline symptoms, infection severity and medical care were administered at enrollment (Table). Controls with no history of SARS-CoV-2 infection were concurrently followed.

Symptom surveys were given at 6 months post-infection. Vaccination status was self-reported. We defined PASC as the presence of one or more symptoms that persisted for at least 4 weeks after acute SARS-CoV-2 infection.

**Table.**
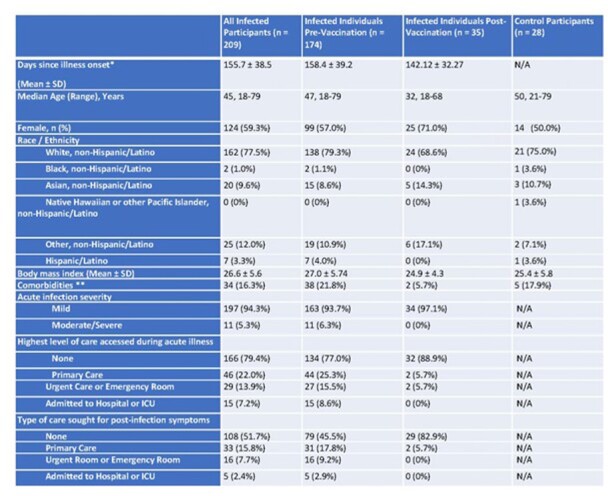
Demographic and Illness Characteristics of Study Participants

**Results:**

Of 369 cases and 93 controls 57% (median age 44.7 years; 59.3% female) and 30% (median age 50.0 years; 50.0% female), completed the 6-month survey, respectively (Table). A total of 174 cases were infected prior to vaccination and 35 were post-vaccination.

A total of 58 (28%) cases reported symptoms at 6 months, compared to 5 (18%) controls (Figure). In participants infected pre-vaccination, 32% reported PASC symptoms, compared to 6% of those infected post-vaccination (Figure; P=0.001).

**Figure:**
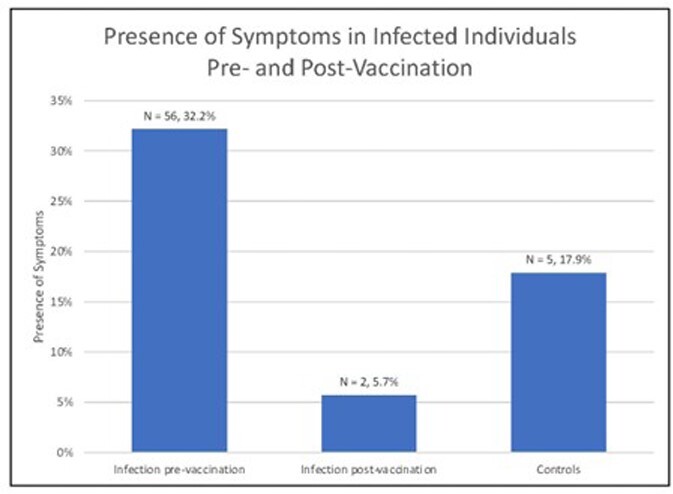

**Conclusion:**

Our study found that the proportion of individuals reporting PASC at 6 months after infection was significantly higher among those infected before SARS-CoV-2 vaccination than those who were infected after. This suggests that timing of vaccination relative to SARS-CoV-2 infection may be associated with the development of PASC symptoms. Symptoms were still reported among many individuals with PASC who were vaccinated after their infection. Further research is required to understand the underlying mechanisms of PASC, and to characterize PASC in those infected after vaccination and with variant of concerns.

**Disclosures:**

**Helen Y. Chu, MD, MPH**, Cepheid: Reagents|Ellume: Advisor/Consultant|Gates Ventures: Grant/Research Support|Merck: Advisor/Consultant|Pfizer: Advisor/Consultant.

**Table.** Demographic and Illness Characteristics of Study Participants

*Days since illness onset refers to the days passed since infection date. **Comorbidities included hypertension, diabetes mellitus, chronic obstructive pulmonary disease, cardiovascular disease, chronic liver disease, chronic kidney disease, HIV, current use of immunosuppressants or diagnosis of cancer.

